# Septic Shock: Management and Outcomes

**DOI:** 10.7759/cureus.32158

**Published:** 2022-12-03

**Authors:** Nojood Basodan, Abdulaziz E Al Mehmadi, Abdullah E Al Mehmadi, Sulaiman M Aldawood, Ashraf Hawsawi, Fahad Fatini, Ziyad M Mulla, Waleed Nawwab, Ammar Alshareef, Amir H Almhmadi, Amin Ahmed, Abdulwahab Bokhari, Abdulaziz G Alzahrani

**Affiliations:** 1 Emergency Department, King Faisal Hospital, Makkah, SAU; 2 Neurosurgery Department, King Faisal Hospital, Makkah, SAU; 3 Orthopedic Department, King Faisal Hospital, Makkah, SAU; 4 Intensive Care Unit, King Faisal Hospital, Makkah, SAU; 5 Internal Medicine Department, King Faisal Hospital, Makkah, SAU

**Keywords:** guidelines, management, diagnosis, sepsis, septic shock

## Abstract

The incidence rates of sepsis and septic shock as a complication have become more common over the past several decades. With this increase, sepsis remains the most common cause of intensive care unit (ICU) admissions and one of the most mortality factors, with a huge burden on healthcare facilities. Septic shock has devastating consequences on patients' lives, including organ failures and other long-term complications. Due to its dynamic clinical presentations, guidelines and tools have been established to improve the diagnosis and management effectively. However, there is still a need for evidence-based standardized procedures for the diagnosis, treatment, and follow-up of sepsis and septic shock patients due to the inconsistency of current guidelines and studies contrasting with each other. The standardization would help physicians better manage sepsis, minimize complications and reduce mortality. Septic shock is usually challenging to manage due to its variety of clinical characteristics and physiologic dynamics, affecting the outcomes. Therefore, this review presented the available data in the literature on septic shock diagnosis, management, and prognosis to have an overview of the updated best practice approach to septic shock.

## Introduction and background

Sepsis is the leading cause of admission to the intensive care unit (ICU) and a major cause of mortality across high-income countries, posing a significant public health burden [1]. In 2009, sepsis was the most expensive healthcare condition in the United States (US), accounting for 5% of total hospital costs [2]. This is due to the fact that sepsis treatment can cost approximately $50,000 per patient. Additionally, studies conducted in the UK estimated that treating severe sepsis would cost the healthcare system roughly £2.5 billion annually [1].

A study conducted in Catalonia between 2008 and 2012 found that sepsis incidence increased annually by 7.3%, from 167.2 per 100,000 people in 2008 to 261.8 per 100,000 people in 2012 [1]. However, the incidence increase rate is estimated to be 9% globally [2]. As of 2017, sepsis was reported to claim 148.1 lives per 100,000 population, with about 8 million deaths each year [3-5]. Its mortality rate varies from 15-30% in high-income countries to 50% or higher in low-income countries [4]. Studies have shown an upward trend in sepsis-related ICU admissions. From 2010-2015, sepsis-related admissions increased from 3.9% to 9.4% in three hospitals in Philadelphia, US, while in New Zealand and Australia, they had increased from 7.2% in 2000 to 11.1% in 2012 [6-7].

Although epidemiological statistics are still lacking, especially in low- and middle-income countries, a recent assessment that sought to determine the global incidence of sepsis found that the overall incidence rate for sepsis and severe sepsis was 288 and 148 per 100,000 people/year, respectively [8]. This review aimed to present the literature data on septic shock to help inform healthcare workers of the most recent best practice and approach to septic shock.

## Review

Clinical features

Severe sepsis and septic shock occur as a result of both community-acquired and healthcare-associated infections. The signs and symptoms include a fever of >38^o^C, shortness of breath, pallor, malaise, diaphoresis, anorexia, respiratory rate of ≥22/min, altered mental status, tachycardia, and hypoxia, among others. Hypothermia < 36^o^C, abnormal breath sounds, and sputum production are found in pneumonia-induced sepsis [9]. The most common initial source, accounting for almost half of all cases, is pneumonia, which could be preceded by urinary tract and intra-abdominal infections [10]. 

Most infections are bacterial in origin, and the most prevalent gram-positive strains are *Staphylococcus aureus* and *Streptococcus pneumoniae*, whereas the most prevalent gram-negative strains are *Escherichia coli*, Klebsiella species, and *Pseudomonas aeruginosa*. However, only a third of blood cultures are usually positive, and cultures from all sites can be negative in a third of cases [1,3,7,10]. The blood culture sensitivity can be lowered by the initiation of empirical antibiotic therapy. Obtaining blood samples before starting antibiotic therapy is recommended to increase the likelihood of positive results [11]. 

The risk for sepsis and septic shock generally increases with age, but it is bimodally distributed, higher in newborns and less in young people, and then the risk rises again after the age of 60 due to immature immunity in newborns and aging immunity in older adults [12,13]. Male gender is associated with an increased risk for sepsis and septic shock. Estrogens have protective effects on immune response and cardiovascular functioning, which might account for the lower risk of sepsis in the female sex [1,13]. Numerous studies have reported no significant risk differences between males and females [1,12-14]. Comorbidities and immunosuppressive medications increase the risk of sepsis and septic shock [1,13]. Sepsis and septic shock can also be influenced by seasons, with higher prevalence in the winter. This is due to increased incidences of pulmonary infections in winter, which are risk factors for sepsis [13]. Furthermore, sepsis and septic shock are linked to other factors like malnutrition, poverty, a lower level of education, the prolonged time between the emergence of symptoms and the start of treatment for sepsis, and infection misdiagnosis [1].

Diagnosis

Septic shock has various signs and symptoms [15]. However, the most important and common one defining septic shock is sepsis (suspected or documented infection) with persistent systolic hypotension of <90mmHg or mean arterial blood pressure of <65mmHg, despite adequate fluid resuscitation and unexplained by other causes and evidence of tissue hypoperfusion [16]. While these signs are commonly present, they should not be required to define shock according to a statement from the European Society of Intensive Care Medicine (ESICM) consensus [16,17]. This is because septic shock has very variable signs and symptoms, depending on which system is affected [10], and it is associated with many conditions, such as surgical site infection [18], trauma [19], burn [20], endocarditis [21], necrotizing fasciitis [22], HIV [23], pancreatitis [24], meningitis [25], septic arthritis [26], and COVID-19 [27].

Tools for sepsis screening were created to encourage early detection of sepsis for timely intervention. Although most of the tools have weak predictive values and have a wide range of diagnostic accuracy, they are still attributed to improved care practices [17]. The tools are used together with clinical symptoms, including systemic inflammatory response syndrome (SIRS) criteria, infection, vital signs, National Early Warning Score (NEWS), Sequential Organ Failure Assessment (SOFA), quick Sequential Organ Failure Score (qSOFA), or Modified Early Warning Score (MEWS) [17,28]. Quick SOFA, or qSOFA, exclusively includes clinical criteria that can be rapidly evaluated in clinical settings, such as level of consciousness, respiratory rate, and systolic blood pressure 100mmHg. The patient is considered to be qSOFA positive when two of these characteristics are present simultaneously [17]. However, the low specificity of qSOFA makes laboratory testing required to differentiate it from other conditions and to assess organ function and acid-base balance [29]. This is because, in most cases, tissue hypoperfusion occurs before the development of hypotension. Despite the clinical assessment, biomarkers are used for diagnosis and outcome prediction. While none are specific enough, they are used in clinical practice to monitor the infectious process or rule out the infection to differentiate between sepsis and other inflammatory disorders. The presence of tissue hypoperfusion can be determined by measuring the blood lactate concentration [29,30]. Therefore, increases in serum lactate levels indicate organ failure or altered clearance progress. Moreover, hyperlactatemia is a serious sepsis marker also utilized as a prognostic indicator since it is linked to a mortality rate rise of 35% to 70% [29].

C-Reactive Protein (CRP)

It is a protein usually increased at the acute phase generated by the liver and other cells, like alveolar macrophages. Bacterial infection is a strong stimulus triggering a marked short-term increase in CRP levels; changes in these levels can help in the diagnosis and prognosis [17]. Therefore, a decline in plasma levels of CRP suggests the clearance of infection and response to antibiotics [29]. However, CRP values do not reflect the acuity as they slowly rise and decrease after several days of treatment, for which CRP is not useful in the early course of sepsis [31,32].

Procalcitonin (PCT)

Procalcitonin is a 116-amino-acid precursor of calcitonin which is secreted in response to toxins. It increases in the first hours after sepsis and reaches a peak in 24-48 hours. The secretion especially increases with systemic bacterial infections and gives minimal differentiation between bacterial and viral infections [29,33]. Normal levels of PCT may exclude the diagnosis. However, PCT is not considered a reason to substitute sepsis. Treatment of sepsis should be initiated even among patients with low levels of PCT [29,30,33].

Cytokines

Cytokines can add more information than PCT or CRP. Interleukin-6 (IL-6), for example, is elevated early after inflammation and correlates with the severity and prognosis. On the other hand, cytokines can elevate in several medical conditions and are not considered a specific biomarker [34].

Presepsin

This is a glycoprotein that functions as a receptor lipopolysaccharide. While having limited diagnostic value, its levels increase early in sepsis compared to PCT. Moreover, levels correlate with the severity and mortality of sepsis, as it was shown that patients with higher presepsin levels have higher mortality rates [35]. Therefore, presepsin is considered a useful biomarker for the management of sepsis as it allows early assessment of severity [35,36].

Monocyte Chemoattractant Protein 1 (MCP-1)

This is a chemokine secreted by many pro-inflammatory cells. Its plasma levels represent a reliable biomarker predicting the prognosis [37,38]. The circulating levels of immunoglobulin (Ig) can give a prediction of mortality in the case of IgG1 [39]. On the other hand, IgG1, IgA, and IgM deficiencies have the greatest effect on survival. Furthermore, Ig levels are useful in indicating the benefits of the administration of intravenous immunoglobulin as a therapy [40].

Pro-Adrenomedullin (Pro-ADM)

It is increased due to the degradation of adrenomedullin among patients with sepsis or septic shock, correlating with the severity of the infection more than PCT and polymerase chain reaction (PCR) [31,32,41]. This enabled the use for the identification of severe cases necessitating admission to the ICU [41,42]. Pro-ADM also helps in the prediction of hospital stay which facilitates the selection of therapy [31,41].

Septic patients management

According to the surviving sepsis campaign guidelines [17, 42-43], septic shock patients should be managed systematically as follows:

Source Control

During the first hours of diagnosis, empiric antibiotic therapy, such as broad-spectrum antibiotics, should be initiated for all suspected patients to help in the source control. If needed, removal of the infected tissues reduces the spread and controls the source of sepsis. 

Management of Shock

The early initiation of management is crucial for survival. Restoring venous pressure to 8-18mmHg, mean arterial pressure to greater than 65, and superior vena cava saturation to 70% are the goals of initial interventions. This is achieved by fluid resuscitation with crystalloid and colloid. Patients may need mechanical ventilation and the use of vasoactive agents such as epinephrine in fluid-refractory cases [44]. Dopamine is not recommended as a first-line agent due to its immunologic dysfunction through lowering prolactin and growth hormone via the inhibitory effect on the hypothalamic-pituitary-adrenal (HPA) axis [45].

Enhancing Host Response

The administration and vasopressin of corticosteroids is indicated in vasoactive-refractory cases or in patients with unstimulated basal cortisol levels [46]. While the administration of the central venous line is not indicated for all cases, it can provide accurate monitoring of mixed venous oxygen (MVO2) and central venous pressure (CVP) [47]. The central line within the right atrium is more accurate than the lower extremity lines [47]. Both dopamine, norepinephrine, and phenylephrine can be safely administered at high doses [48]. Early goal-directed therapy (EGDT) is a standard practice in the management of severe sepsis; however, this approach has been shown to confer survival rates [49]. Survival also can be influenced by the stabilization of blood pressure [50]. 

Antimicrobial Therapy

The empirical broad-spectrum antibiotic should be administrated within the first hour. However, timing is controversial since it has not been established by evidence base protocols [9]. Covering most pathogens with multi drugs regimens is important to ensure sufficient coverage. The initial choice should be based on individual factors affecting antibiotic effectiveness, such as patient age, source of infection, previous antibiotic use, comorbidities, multi-drug resistant organism, the severity of the septic shock, and patient immunity [9,43]. Adequate dosing is also important, as drug efficacy depends on peak blood level and minimum inhibitory concentration of the pathogens. Thus, an initial higher dose is recommended to maintain the drug's therapeutic blood level [9].

Complications

Sepsis-Associated Lung Dysfunction

Sepsis is commonly accompanied by lung dysfunction, also known as acute respiratory distress syndrome (ARDS) or acute lung injury (ALI) [51]. According to a prior study, sepsis-related ARDS had a worse prognosis than non-sepsis-related ARDS [52]. Patients with sepsis-induced ARDS have a higher case fatality rate than those with other ARDS risk factors [53]. The basic pathophysiology of ARDS is an increase in microvascular permeability brought on by dysregulation of cell-to-cell communication or tissue death. Numerous studies have shown that neutrophils, which are important terminal effector cells in innate immunity, significantly contribute to tissue harm in ARDS [54]. It has been demonstrated that neutrophils also contribute to the malfunctioning of other organs. Granular enzymes released by neutrophils, reactive oxygen metabolites, bioactive lipids, and cytokines can all lead to the development of neutrophil extracellular traps, the majority of which can either directly or indirectly harm tissues, increasing microvascular permeability and cause pulmonary edema [51,55]. A powerful neutrophil chemotactic chemokine known as interleukin 8 also plays significant roles in the etiology of ARDS, in addition to tumor necrosis factor (TNF) and IL-1, which are significant contributors to septic shock [55]. Some damage-associated molecular patterns (DAMPs) are now understood to operate as mediators or cytokines. High mobility group box 1 protein (HMGB-1), a molecule initially classified as a nuclear binding protein, has been linked to sepsis and sepsis-associated ARDS [56]. A sepsis-like condition and ALI can be brought on by mitochondrial DAMPs, which can also trigger polymorphonuclear leukocytes (PMNs). Apoptosis and autophagy are also involved in sepsis-induced tissue damage related to ARDS and neutrophil-mediated tissue injury [51]. 

Cardiac Dysfunction

Following the use of vasopressors or volume resuscitation, venous return increases, and patients adopt a hyperdynamic profile with high cardiac output and low systemic vascular resistance. However, a lowered myocardial function frequently coincides with this reaction [51]. IL-1 and IL-6 are pro-inflammatory cytokines that decrease the contractility of cardiomyocytes and cause the coronary endothelium to produce vascular cell adhesion molecule 1 (VCAM-1), facilitating the migration of neutrophils into the myocardium [51,54]. Importantly, nitric oxide (NO) promotes the release of pro-inflammatory cytokines, decreases myocardial oxygen use, and downregulates beta-adrenergic receptors. As a result, nearly one in three septic patients have reversible left ventricular systolic dysfunction caused by hypokinesia and a lower ejection fraction, with unknown implications for survival [6,51]. On the other side, one out of two patients has left diastolic dysfunction, which is linked to an 80% higher risk of passing away. The risk of death is increased by 60% in septic patients with right ventricular dysfunction [51].

Kidneys

Another frequently targeted organ in this gradual organ dysfunction is the renal system. More than half of sepsis or septic shock patients develop acute kidney injury (AKI) [54]. AKI is defined as an increase in serum creatinine levels of less than 0.3 mg/dl in 48 hours, an increase in urine output of more than 0.5 ml/kg/h for more than six hours, or a 50% increase from baseline in seven days [57]. Compared to non-sepsis-associated AKI, patients with sepsis-associated AKI have a 62% vs. 36% higher risk of in-hospital mortality [58,59]. However, the fundamental processes of sepsis-associated AKI are not fully known despite its high prevalence. The paradigm has been renal hypoperfusion leading to acute tubular necrosis. tubular apoptosis and oxidative stress [60,61]. Additionally, the management of sepsis can also cause AKI by using nephrotoxic medications and excessive or non-physiological fluid resuscitation [59]. Increased central venous pressure causes renal vascular pressure to rise, which in turn causes organ edema, elevated intracapsular pressure, and a decrease in glomerular filtration rate [57,58,61]. Evidence suggests the use of normal saline has been linked to renal damage and poor survival than Ringer lactate [59].

Liver

The mortality rate for patients with sepsis/septic shock complicated by liver failure is significantly high [62]. Sepsis-related liver impairment is a very complex and poorly understood etiology. The surviving sepsis campaign guidelines state that a rise in bilirubin concentration >2mg/dL and the onset of coagulation problems with an international normalized ratio (INR) of >1.5 are indicators of liver dysfunction during sepsis [43]. Bilirubin is ineligible to serve as a single metric to reflect the complex liver functions due to its lack of specificity and inability to distinguish between acute liver failure and earlier liver dysfunction [62,63]. Hypoxic hepatitis, sepsis-induced cholestasis, and disruption of protein synthesis, such as coagulopathies, are clinical symptoms of sepsis-associated liver dysfunction [62]. Analgosedation used in the critical care unit may mask the symptoms of detoxifying liver malfunction, which is linked to an increase in blood ammonia content and manifests as confusion, loss of consciousness, and hepatic encephalopathy [62]. Even though septic shock is a complication of sepsis, it can lead to serious and life-threatening complications, such as disseminated intravascular coagulation (DIC) from systemic intravascular coagulation activation caused by various factors [64]. This coagulation abnormality is a significant and common complication in septic shock patients. In addition, hemostatic system dysregulation can result in DIC, microvascular thrombosis, hypoperfusion, major organ dysfunction, and death. Furthermore, despite using universal, guideline-recommended thromboprophylaxis, patients in septic shock have a high incidence of venous thromboembolism (VTE). Patients with sepsis with clinically significant VTE had a significantly longer ICU length of stay than patients who did not have VTE [65].

Prognosis

Septic shock is the most feared progression of sepsis due to its higher case mortality rate. Mortality is also dependent on age, organism, therapy, and organ failure [66]. Higher mortalities correlate to SIRS. On the other hand, sepsis affects the quality of life among those who survive after hospital discharge [66-67]. Septic cases are usually seen in the emergency department, and admission to a ward or ICU, initiation of therapy, and ventilation greatly influence the outcome of the disease and hospital stay [42]. Patients who survive a prolonged period of ICU care for sepsis usually face a long and difficult road to recovery. Sepsis, in general, has high readmission rates, a longer hospital stay, post-discharge hospice, and impaired quality of life [17]. Readmission within three months is estimated to be among 40% of sepsis survivors causing higher costs. Survivors are prone to AKI, cardiovascular events, and recurrence [42,68]. Advanced age requires a follow-up within the first week of discharge which can be done in primary care settings [69-70]. 

## Conclusions

Septic shock remains a significant cause of death in critically ill patients. ARDS is also the deadliest complication of severe sepsis, with a high mortality rate. The availability of diverse modalities helps accurately diagnose and manage sepsis and septic shock patients. Good outcomes rely on early diagnosis and prompt interventions. Delays, on the other hand, will result in severe and long-term complications, including death. To prevent acute and long-term complications, treating septic shock patients usually involves a multidisciplinary approach consisting of clinicians with various expertise, physiotherapists, psychologists, surgeons, etc., leading to restoration of function within three months for most septic shock survivors. Thanks to advancements in training, improved surveillance and monitoring, and prompt initiation of therapy to treat the underlying infection and support failing organs, mortality and morbidities can decrease. More standardized guidelines should be constantly developed and updated based on the most recent data to guide sepsis and septic shock management using evidence-based best-practice approaches. This would help reduce mortality and morbidity and relieve the burden posed by this public health problem.
